# Supplementary material for: Evidence supported by Mendelian randomization: impact on inflammatory factors in knee osteoarthritis
Source: Front Med (Lausanne). 2024 May 28;11:1382836. doi: 10.3389/fmed.2024.1382836 (PMC11165061; doi:10.3389/fmed.2024.1382836)
Supplement: Supplementary file 1 [file Data_Sheet_1.ZIP › data/GCST90274832_5e6/832.forest.pdf]

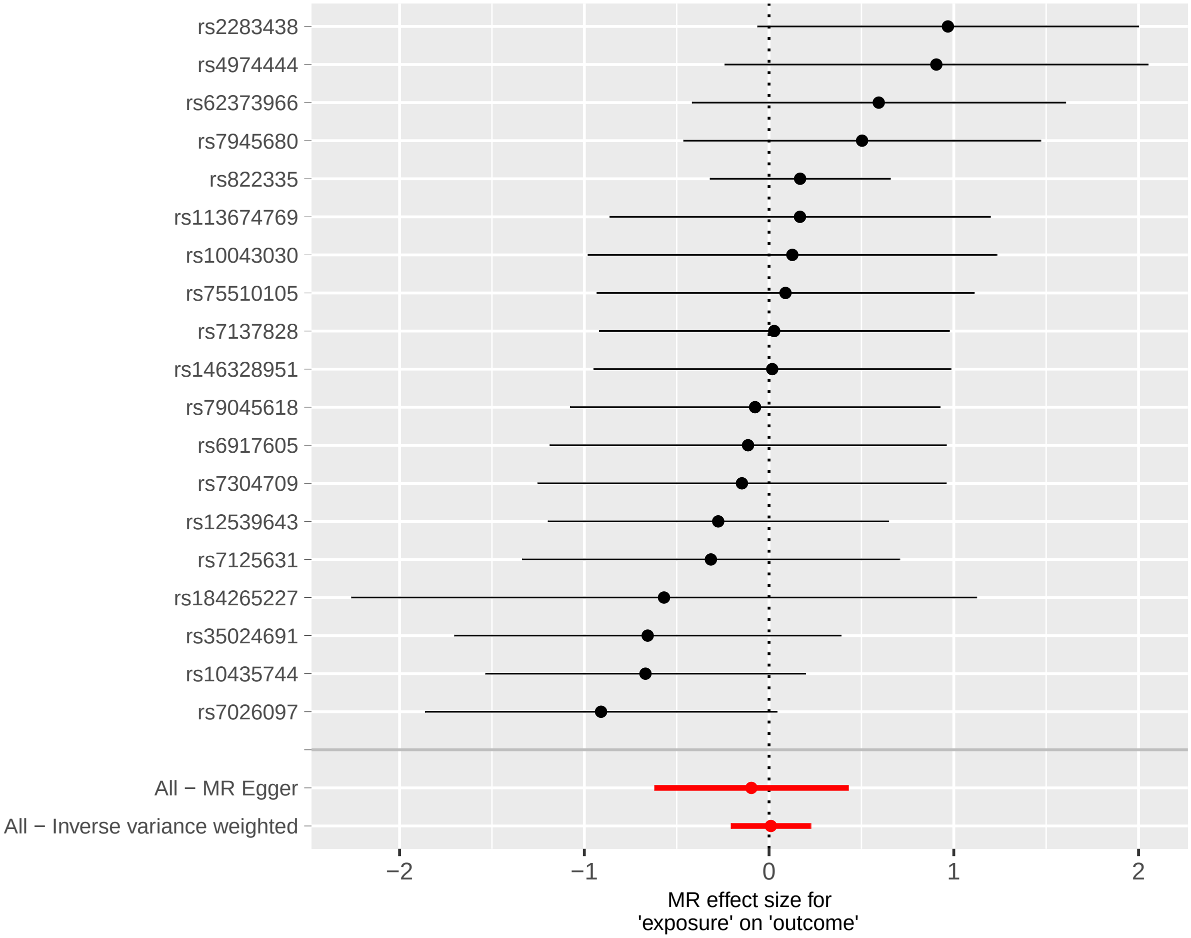
rs2283438
rs4974444
rs62373966
rs7945680
rs822335
rs113674769
rs10043030
rs75510105
rs7137828
rs146328951
rs79045618
rs6917605
rs7304709
rs12539643
rs7125631
rs184265227
rs35024691
rs10435744
rs7026097
All − MR Egger
All − Inverse variance weighted
−2
−1
0
1
2
MR effect size for
'exposure' on 'outcome'
